# GENIE: a software package for gene-gene interaction analysis in genetic association studies using multiple GPU or CPU cores

**DOI:** 10.1186/1756-0500-4-158

**Published:** 2011-05-26

**Authors:** Satish Chikkagoudar, Kai Wang, Mingyao Li

**Affiliations:** 1 Department of Biostatistics and Epidemiology, University of Pennsylvania School of Medicine, Philadelphia, PA, USA; 2 Zilkha Neurogenetic Institute, Keck School of Medicine, University of Southern California, Los Angeles, CA, USA; 3 Department of Psychiatry, Keck School of Medicine, University of Southern California, Los Angeles, CA, USA; 4 Department of Preventive Medicine, Keck School of Medicine, University of Southern California, Los Angeles, CA, USA

## Abstract

**Background:**

Gene-gene interaction in genetic association studies is computationally intensive when a large number of SNPs are involved. Most of the latest Central Processing Units (CPUs) have multiple cores, whereas Graphics Processing Units (GPUs) also have hundreds of cores and have been recently used to implement faster scientific software. However, currently there are no genetic analysis software packages that allow users to fully utilize the computing power of these multi-core devices for genetic interaction analysis for binary traits.

**Findings:**

Here we present a novel software package GENIE, which utilizes the power of multiple GPU or CPU processor cores to parallelize the interaction analysis. GENIE reads an entire genetic association study dataset into memory and partitions the dataset into fragments with non-overlapping sets of SNPs. For each fragment, GENIE analyzes: 1) the interaction of SNPs within it in parallel, and 2) the interaction between the SNPs of the current fragment and other fragments in parallel. We tested GENIE on a large-scale candidate gene study on high-density lipoprotein cholesterol. Using an NVIDIA Tesla C1060 graphics card, the GPU mode of GENIE achieves a speedup of 27 times over its single-core CPU mode run.

**Conclusions:**

GENIE is open-source, economical, user-friendly, and scalable. Since the computing power and memory capacity of graphics cards are increasing rapidly while their cost is going down, we anticipate that GENIE will achieve greater speedups with faster GPU cards. Documentation, source code, and precompiled binaries can be downloaded from http://www.cceb.upenn.edu/~mli/software/GENIE/.

## Background

The advent of high-throughput genotyping technologies has made it possible to study human genetic variation on a genome-wide scale. Recent years have seen an explosion of results generated from genome-wide association studies (GWAS). Most GWAS focus on single marker-based analysis in which each marker is analyzed individually, ignoring the dependence or interactions between markers. Although this approach has led to the discovery of disease susceptibility genes for many diseases, the identified markers often only explain a small fraction of the phenotypic variation, suggesting a large number of disease variants are yet to be discovered. It is becoming increasingly evident that gene-gene interactions play an important role in the etiology of complex diseases and traits [1-3], and likely explain some fraction of the "missing heritability". Gene-gene interaction is often studied using a regression framework in which a pair of SNPs and their interaction terms are included as predictors. The drawback of such analysis is that the number of tests will be extremely large. For example, in the case of a GWAS with 500,000 SNPs the number of SNP pairs to be studied amounts to ~125 billion. The running time quickly becomes an issue due to the large number of pairs.

However, gene-gene interaction analysis is parallelizable in nature. Most of the current Central Processor Units (CPUs) have multiple cores. Parallel computing until recently meant using a computing cluster having multiple nodes with multi-core CPUs. The costs of building a computing cluster may run in hundreds of thousands of dollars, making it cost prohibitive. An emerging economic scientific computing paradigm is to use Graphics Processing Units (GPUs) that are present in graphic cards of most desktop computers or workstations for general purpose computing. A GPU is a processing unit that was traditionally used for accelerating graphical operations. The power of GPUs has been used to implement faster software solutions for biological problems [4-8]. For example, Schupbach et al. [8] developed a GPU-based software package that greatly speeds up gene-gene interaction analysis of quantitative traits.

A typical graphics card has several processors as well as its own dedicated memory. We will be using the term "device memory" to refer to the built-in memory of the graphics card in the rest of this paper. Several vendors of graphics cards offer architectures and programming tools that enable GPU-based general purpose computing using high level programming language extensions. NVIDIA Common Unified Device Architecture (CUDA) is an example of a graphics card architecture for parallel general purpose computing. CUDA follows the Single Instruction Multiple Thread (SIMT) architecture that is similar to the Single Instruction Multiple Data (SIMD) architecture of parallel computing. In the case of CUDA, this means that multiple threads on the same instructions are executed simultaneously on different data. Programmers can exploit the CUDA architecture with relative ease to solve larger problems that can be decomposed into several sub-problems that can be solved in parallel. The computing power offered by the latest graphic cards is comparable to that of a computing cluster with hundreds of CPUs, but the GPU programming approach for parallel computing is much cheaper than using a traditional computing cluster.

**Figure 1:**
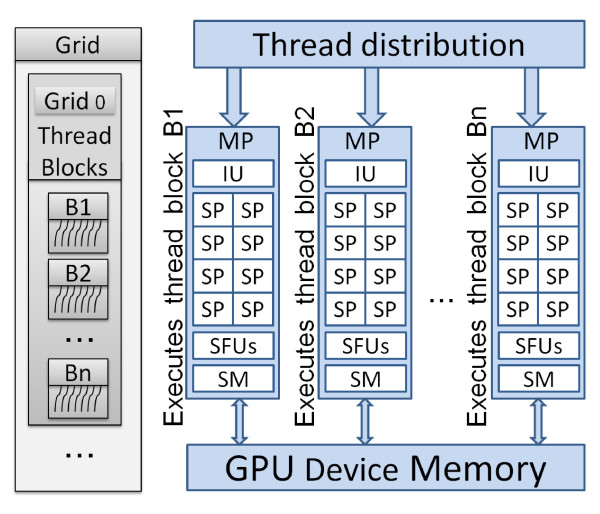
**NVIDIA GPU Architecture**. Simplified GPU Architecture: The grey rectangles of thread blocks are for illustration purposes and are not a physical part of the architecture. MP = Multi Processor, SM = Shared Memory, SFU = Special Functions Unit, IU = Instruction Unit, SP = Streaming processor (core).

CUDA compatible graphics cards have several processors that are also known as multiprocessors (MP) that in turn have several stream/thread processors (SP) known as cores (Figure 1). CUDA arranges threads in grids and blocks. A block is a collection of threads, while a grid is a collection of blocks. CUDA allows the sizes of the blocks and grids to be manipulated programmatically. There is a limit to the maximum number of threads that can be present in a block. This limitation exists because a block has to reside on a single MP and share that MP's resources. On a Tesla C1060 the maximum number of threads per block is 512. We use the term CUDA thread or GPU thread interchangeably in this paper.

**Figure 2:**
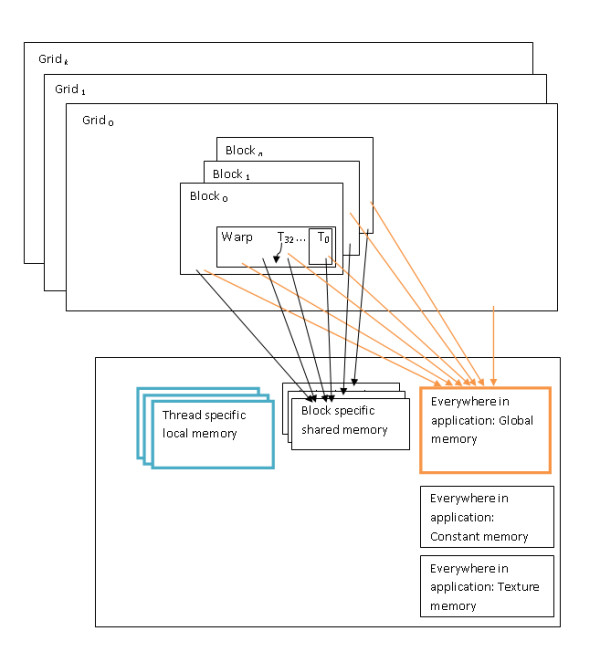
**CUDA Thread and Memory Hierarchy**. The dashed rectangle at the top half of the diagram shows the CUDA thread hierarchy while the large rectangle in the lower half shows the hierarchy of memory spaces in a CUDA device. T0 to T32 represent the threads contained in a warp. The current version of GENIE uses the global and local memory spaces that are represented by the orange and aqua colored boxes respectively.

CUDA provides a new scalable parallel programming model along with an instruction set. CUDA also provides several levels of abstraction in terms of threads and memory hierarchies. Such abstractions help CUDA provide data parallelism and thread parallelism. Programmers are exposed to these abstractions in the form of API. Figure 2 shows the CUDA memory and thread hierarchies. A kernel is a C function that is executed by several GPU threads in parallel. A program exploiting the power of GPUs generally has portions of code that runs on the CPU as well as calls to kernels that run on the GPU cores. The CUDA runtime system is aware of the system details of the CUDA capable graphics card such as the number of MPs and cores. The runtime system schedules a thread block to be executed on the next available MP. The parallel threads in a block are executed by the assigned MP in groups of 32 known as warps. Thread blocks can be scheduled to execute independent of each other over any number of GPU cores. Each CUDA thread has local memory that is limited to 16 KB in case of a C1060 graphics card. As shown in Figure 2, global memory is accessible by every thread. Each block of threads has access to a shared memory.

Currently, there are no software packages that directly address the gene-gene interaction analysis problem for binary traits. Moreover, there are no gene-gene interaction analysis software packages that use multiple processor cores present in CPUs or GPU cards. Our software package, GENIE, utilizes multiple cores to parallelize gene-gene interaction analysis. As we show in the Results, GENIE achieves ~27 times speedup over a single-core CPU, and making large-scale gene-gene interaction analysis feasible even on a desktop computer.

## Implementation

### Development environment

GENIE was implemented using C language and CUDA C language extension. It was developed on a Dell Precision T7500 Windows XP workstation that has 4 GB of main memory, a quad-core 2.13 GHz Intel Xeon processor, an NVIDIA Quadro FX1800 graphics card, and an NVIDIA Tesla C1060 graphics card with 4 GB of device memory. The NVIDIA C1060 card has 30 MPs and 240 cores. It has a CUDA compute capability of 1.3. We used CUDA toolkit version 3.1 and MS Visual C++ 2008 to compile the program under windows. To run GENIE, we used the NVIDIA Tesla C1060 card, whereas the Quadro FX1800 card was used only for display purposes.

### Data Pre-processing

GENIE assumes the input genetic association study files contain case/control data with genotypes encoded under an additive model. Users can use the data converter tool that is a part of the GENIE software package to convert their data files to GENIE format. GENIE documentation includes detailed instructions and step-by-step examples that illustrate this process.

### Logistic Regression

The interaction between a pair of SNPs is tested using a logistic regression framework in which the SNP genotypes and their interaction terms are included as predictors. The model is expressed as the following:(1)

where *Y *(1: case; 0: control) is the disease status, and *g*_*j *_(= 0, 1, and 2) is the genotype score that counts the number of minor alleles at SNP *j*. Interaction between the two SNPs is assessed by testing H_0_: *β*_12 _= 0 using a likelihood ratio test, which is asymptotically distributed as Chi-squared distribution with one degree of freedom. We note that this interaction test is implemented PLINK (http://pngu.mgh.harvard.edu/purcell/plink/) [9], a popular software package for whole-genome data analysis. PLINK analyzes one SNP pair at a time despite that the analysis of all SNP pairs are parallelizable in nature.

### Parallelization Algorithm

We propose to parallelize the interaction analysis of different SNP pairs. Specifically, the GENIE algorithm partitions the input dataset into equally sized non-overlapping fragments with up to *S *SNPs each. This gives GENIE the ability to handle large datasets and maintain a small memory footprint in terms of output variables. We refer to *S *as the fragment size. As mentioned earlier, the interaction between a pair of SNPs is tested using a logistic regression framework. We briefly describe the algorithm below:

1) Read the genetic association study data file. Let fragment size *S *be a non-zero integer.

2) Partition the data into non-overlapping fragments containing of *S *SNPs each. Let us assume that the fragments are numbered from 1 to *F*, where *F *is the total number of fragments. The partitioning of data is done in memory and no physical files need to be generated.

3) For each fragment *i*, where 1≤ *i *≤ *F:*

a. Test the interaction of *S*(*S *-1)/2 SNP pairs within fragment *i *in parallel.

b. For each of the other fragment *j *>*i*, test the interaction of *S*^2 ^SNP pairs across fragment *i *and fragment *j *in parallel.

c. Output only those results that meet a prespecified *P*-value threshold.

The fragment size *S *translates to the maximum *x *or *y *dimension of a thread block, which is 512 for cards with a compute capability of less than 2.0. As mentioned earlier, the size of a full warp is 32. We follow NVIDIA's performance recommendations [10,11] and recommend that *S *must be a non-zero integer multiple of 32 that is less than or equal to 512.

The interaction analysis for each SNP pair is run on a separate GPU thread. The GPU kernel function of GENIE implements logistic regression. We modified PLINK's Newton's algorithm to fit the logistic regression model in (1). Specifically, we unrolled loops in the CUDA kernel. CUDA single precision floating point fast math functions were used in the CUDA kernel implementation. Due to the size of the input dataset, the current implementation of the GENIE algorithm makes use of local and global memory spaces. The input dataset as well as the intermediate output are stored in the global memory. We do not utilize any constant memory, shared memory, and texture memory spaces of the GPU device. We minimize the local memory utilization of the interaction analysis module/kernel to facilitate parallelization. Each GPU thread utilizes a maximum of 16 KB of the local memory. We use a single grid of two dimensional thread blocks. We enabled compiler code optimization. In order to reduce memory latency, we restrict the number of data transfers between main and device memories. Initially, GENIE reads the entire dataset into the main memory of the computer and then transfers it to the global memory space of the device memory. Intermediate results are periodically transferred from the global memory space of the device memory to the main memory of the workstation and then written to the output file. We also reduced the number of registers that are used by each kernel using the -maxrregcount compiler option in order to improve the CUDA utilization/occupancy.

Due to CUDA local memory limitations of the NVIDIA Tesla C1060 card, we set the maximum number of individuals in a genetic association studies to 4,000, but this number can be increased for cards with larger local memory. When the total number of SNPs is not a multiple of *S*, the last fragment has less than *S *total SNPs in it. In this case, we pad the rest of the fragment with missing values to maintain a fragment size of *S*.

The algorithm presented above can be easily extended to support multiple CPU cores. We implemented a simple serial single threaded version of the GENIE algorithm. We support multiple-cores using a script that first splits the input dataset into as many parts as there are cores. It then invokes a separate run of single threaded GENIE on each separate subset or part of the dataset.

### Software Capabilities

The current version of GENIE is a command line program and has a number of user input options. GENIE can be run in a GPU mode or in a CPU mode. The GENIE software package includes a GENIE executable, a data converter utility executable, and a script file. Multiple GPU cards, GPU clusters, multi-core CPUs, and CPU clusters are supported using the script file. This enables GENIE to handle datasets generated from larger genetic association studies in a scalable fashion. In the GPU mode, the interaction analysis is carried out using a CUDA compatible graphics card. If a machine has several GPU cards, GENIE allows users to choose a card that will be used to run the analysis. The script file can be used to distribute the analysis task across multiple GPU cards.

In the CPU mode, the interaction analysis is carried out using a single processor core. If a machine has a multi-core CPU, the script file provided with GENIE package can be used to distribute the analysis task across multiple cores. Currently, the script distributes fragments among several separate parallel runs of a single threaded GENIE implementation for CPUs.

### Findings

Data from the University of Pennsylvania High-Density Lipoprotein Cholesterol (HDL-C) Study was used to test GENIE. In this study, subjects of European ancestry with HDL-C > 90^th ^percentile for age and gender were considered as cases and subjects with HDL-C < 30^th ^percentile for age and gender were considered as controls. 625 cases and 606 controls were genotyped using the IBC 50K SNP array [12]. We extracted 23,470 SNPs that had a Minor Allele Frequency (MAF) greater than 0.15 for interaction analysis.

**Figure 3:**
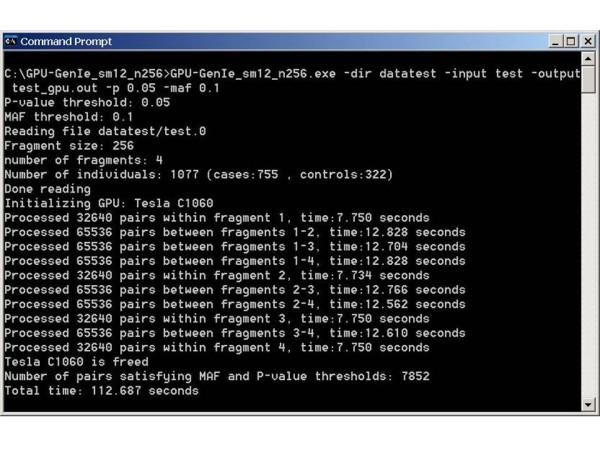
Screenshot of a test run of GENIE

**Figure 4:**
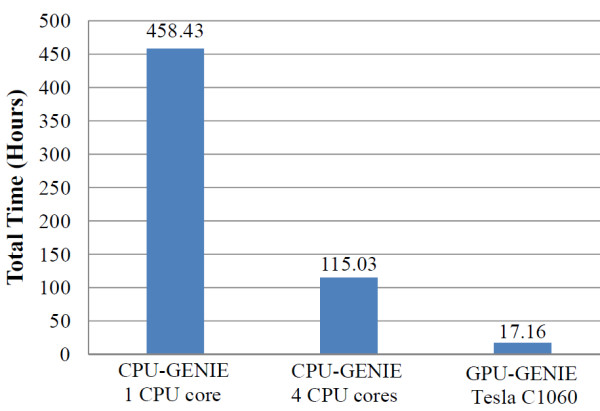
**GENIE execution time on the HDL study dataset**. GPU-GENIE achieves a speedup of 6.7 fold over CPU-GENIE using 4 Intel Xeon cores and 26.7 fold over CPU-GENIE using a single Intel Xeon core.

We tested GENIE on the same workstation that was used for its development. A fragment size of 256 was used during testing. Figure 3 shows the screenshot of a sample run of GENIE. We call the CPU mode runs of GENIE as CPU-GENIE and the GPU mode runs of GENIE as GPU-GENIE for comparison purposes. Speedup was calculated as the ratio of total time taken by the interaction module of CPU-GENIE and the total time taken by the interaction module of GPU-GENIE. The CPU-GENIE script was used to run CPU-GENIE using all four CPU cores. In this case, the script distributed fragments among four separate parallel runs of a single threaded CPU-GENIE implementation. The total time taken by CPU-GENIE with a single core was calculated as a sum of the execution times for each of the four CPU-GENIE runs. Figure 4 shows the execution times of CPU-GENIE and GPU-GENIE. It took CPU-GENIE 458 hours to analyze the HDL-C dataset using a single Intel Xeon CPU core and 115 hours using all four Intel Xeon CPU cores. In contrast, GPU-GENIE achieves a speedup of almost 27 over the single CPU core run of CPU-GENIE and finishes the analysis in around 17 hours. To match the performance of GPU-GENIE, CPU-GENIE requires a CPU cluster with at least 27 processor cores.

The GENIE output file contains 148,590 pairs that satisfy the MAF threshold of 0.15 and P-value threshold of 0.001. Table 1 shows a subset of the GENIE interaction analysis results. Interestingly, all SNP pairs with P-value < 10^-9 ^involve rs1864163, a SNP in gene *CETP *that encodes for cholesteryl ester transfer protein. It is well known that *CETP *promotes the transfer of cholesteryl esters from HDL to low-density lipoprotein, and individuals that are genetically deficient for *CETP *often have extremely high HDL levels. Various genetics studies have shown that *CETP *SNPs are significantly associated with HDL-C level. In our analysis, we found that *CETP *may also interact with other genes in regulating the level of HDL-C.

**Table 1:** Subset of the results produced by GENIE for the HDL-C study showing SNP pairs having an interaction P-value less than 10^-8^.

Gene (SNP1)	Gene (SNP2)	SNP1	SNP2	P-Value
*CETP*	*HDAC4*	rs1864163	rs3791373	7.87 × 10^-11^
*CETP*	*BCL2*	rs1864163	rs1982673	1.53 × 10^-10^
*CETP*	*SCO1*	rs1864163	rs9897641	2.10 × 10^-10^
*CETP*	*ABCC1*	rs1864163	rs215100	3.09 × 10^-10^
*CETP*	*BCL2*	rs1864163	rs2046137	4.66 × 10^-10^
*CETP*	*BCL2*	rs1864163	rs1531697	8.91 × 10^-10^

## Discussion

Recognizing the ongoing debate regarding using GPUs vs. CPUs for general purpose computing [13], we have developed a software package for genetic interaction analysis that takes advantage of multiple cores present in GPU cards and multi-core CPUs. Clusters containing nodes with multiple CUDA based graphics cards are also available for GPU computing purposes. Although we presented our results only for one GPU card, GENIE can easily handle multiple GPU cards and GPU clusters using a script included with the GENIE package. This is possible because GENIE works on partitions of a genetic association study dataset in a decoupled fashion.

The GENIE source code is optimized for the underlying GPU card and CPU. GENIE was implemented by following the NVIDIA CUDA C best programming practices [10]. To make it easily parallelizable, we also minimized the amount of local memory used by the interaction analysis module. As documented by several CUDA publications [10,11,14], CUDA implementations perform better when all the cores are utilized. Underutilization of the cores can make CUDA implementations perform significantly worse than serial CPU based implementations [10,11,13].

**Figure 5:**
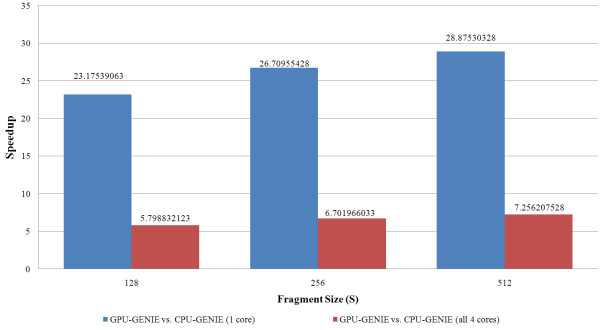
**A study of the effect of fragment size (S) on GPU-GENIE's speedup for the HDL study dataset**. Fragment size has an effect on the amount of speedup that is achieved by GPU-GENIE over CPU-GENIE. The largest speedup is attained for S = 512. As mentioned earlier, these experiments were carried out using a quad core Intel Xeon processor and an NVIDIA C1060 graphics card.

The speedup achieved by the GPU mode of GENIE over its CPU implementation depends on the power of the graphics card and the fragment size *S*. Figure 5 shows the effect of various fragment sizes on the speedup. The optimal value of the fragment size *S *can vary depending upon the graphics card that is used to run GENIE. The GPU computing field is growing rapidly. Faster and cheaper cards are being frequently released. NVIDIA recently released a faster CUDA based architecture called Fermi. The NVIDIA Tesla C2050 is an example of a Fermi based graphics card. A higher GPU mode speedup can be achieved by making use of the latest GPU computing capable graphics cards.

The NVIDIA Tesla C1060 card that was provided by Dell with our workstation cost us $1,700, but it can be purchased online for a significantly cheaper price. The total cost of the workstation was $3,500. A CPU cluster with at least 27 processor cores is needed to match GPU-GENIE's performance. If each machine has a quad-core processor and costs $3,000, a CPU cluster based solution would cost at least $21,000. Thus, GPU computing offers an economical solution to computationally intensive problems.

Future work will involve utilizing Message Passing Interface (MPI) to make GENIE's support of multiple graphics cards, GPU computing clusters, multi-core CPUs and CPU clusters more efficient. Since CUDA code can be easily ported to OpenCL using OpenCL translators such as Swan [15], we plan to use OpenCL to implement a graphics card vendor independent implementation of GENIE. We also plan to implement a webserver version of GENIE.

## Conclusions

GENIE is user friendly and is scalable and can handle large datasets as long as they fit the memory of the graphics card. As GPU cards with bigger memory capacities are entering the market at ever cheaper prices, it is now possible to analyze entire GWAS datasets economically with GENIE. An advantage of running GENIE in GPU mode is that a bulk of the computation is handled by the GPUs and the CPU is free to be utilized by other programs. Although GENIE was originally developed for GPU card, we have provided script that allows the users to run GENIE using CPUs. If the users do not have access to a GPU card, but have access to a CPU cluster, GENIE can still be easily used to perform interaction analysis.

## Availability and requirements

GENIE is implemented using C and NVIDIA Common Unified Device Architecture (CUDA) C extension. The current version of GENIE is built for the windows operating system. GENIE needs to be recompiled to run on Unix and Linux. We recommend that users run GENIE on a CUDA compatible graphics card such as NVIDIA Tesla C1060 or better. NVIDIA recommends using a dedicated graphics card to perform GPU based computing, which means that all display related activities must be carried out using an additional graphics card. Documentation, source code, and precompiled binaries can be downloaded from http://www.cceb.upenn.edu/~mli/software/GENIE/.

## Competing interests

The authors declare that they have no competing interests.

## Authors' contributions

SC, KW and ML designed the study. SC conducted the experiment and implemented the software package. SC and ML wrote the manuscript. All authors have read and approved the final manuscript.
